# Tumor-Infiltrating Neutrophils after Neoadjuvant Therapy are Associated with Poor Prognosis in Esophageal Cancer

**DOI:** 10.1245/s10434-022-12562-5

**Published:** 2022-10-02

**Authors:** Carlos S. Cabalag, Owen W. J. Prall, John Ciciulla, Laurence A. Galea, Niko Thio, Madawa Jayawardana, Trishe Y. M. Leong, Julia V. Milne, Kenji M. Fujihara, Lynn Chong, Michael W. Hii, Gisela Mir Arnau, Paul J. Neeson, Wayne A. Phillips, Cuong P. Duong, Nicholas J. Clemons

**Affiliations:** 1 Cancer Research, Peter MacCallum Cancer Centre, Melbourne, VIC Australia; 2 Cancer Surgery, Peter MacCallum Cancer Centre, Melbourne, VIC Australia; 3 Sir Peter MacCallum Department of Oncology, University of Melbourne, Parkville, VIC Australia; 4 Department of Pathology, Peter MacCallum Cancer Centre, Melbourne, VIC Australia; 5 Department of Anatomical Pathology, Melbourne Pathology, Sonic Healthcare, Melbourne, VIC Australia; 6 Department of Anatomical Pathology, St Vincent’s Hospital, Fitzroy, VIC Australia; 7 Department of Surgery (St Vincent’s Hospital), University of Melbourne, Fitzroy, VIC Australia; 8 Department of Upper GI and Hepatobiliary Surgery, St Vincent’s Hospital, Fitzroy, Victoria Australia; 9 Department of Clinical Pathology, University of Melbourne, Parkville, VIC Australia

## Abstract

**Background:**

In esophageal cancer (EC), there is a paucity of knowledge regarding the interplay between the tumor immune microenvironment and response to neoadjuvant treatment and, therefore, which factors may influence outcomes. Thus, our goal was to investigate the changes in the immune microenvironment with neoadjuvant treatment in EC by assessing the expression of immune related genes and their association with prognosis.

**Methods:**

We examined the transcriptome of paired pre- and post-neoadjuvant treated EC specimens. Based on these findings, we validated the presence of tumor-infiltrating neutrophils using CD15^+^ immunohistochemistry in a discovery cohort of patients with residual pathologic disease. We developed a nomogram as a predictor of progression-free survival (PFS) incorporating the variables CD15^+^ cell count, tumor regression grade, and tumor grade.

**Results:**

After neoadjuvant treatment, there was an increase in genes related to myeloid cell differentiation and a poor prognosis associated with high neutrophil (CD15^+^) counts. Our nomogram incorporating CD15^+^ cell count was predictive of PFS with a C-index of 0.80 (95% confidence interval [CI] 0.68–0.9) and a concordance probability estimate (CPE) of 0.77 (95% CI 0.69–0.86), which indicates high prognostic ability. The C-index and CPE of the validation cohort were 0.81 (95% CI 0.69–0.91) and 0.78 (95% CI 0.7–0.86), respectively.

**Conclusions:**

Our nomogram incorporating CD15^+^ cell count can potentially be used to identify patients at high risk of recurrent disease and thus stratify patients who will benefit most from adjuvant treatment.

**Supplementary Information:**

The online version contains supplementary material available at 10.1245/s10434-022-12562-5.

Currently, curative-intent treatment for locally advanced esophageal cancer (EC) includes neoadjuvant treatment with chemoradiotherapy or chemotherapy, followed by surgical resection.^1^ Despite multimodality, 5-year progression-free survival (PFS) remains poor; more than 50% of patients succumbing to metastatic disease.^1^

Over the past two decades, numerous studies have investigated the tumor–immune microenvironment in the search for biomarkers that may explain the mechanisms of treatment resistance, immune-evasion, and metastasis. In EC, the tumor–immune microenvironment is largely immunosuppressive and pro-tumorigenic, characterised by an exhausted adaptive immune response in addition to a predominant infiltration by neutrophil-like, myeloid-derived suppressor cells (MDSC).^2^ Research into the effects of chemotherapy and/or radiation on the tumor–immune microenvironment have largely concluded that these treatments have immunostimulatory and abscopal effects, which lead to an immunogenic tumor cell death response mediated by T-cell priming and antigen presentation.^3^ However, emerging evidence suggests that subjecting solid tumors with a preexisting, immunosuppressive microenvironment to neoadjuvant treatment may, in fact, further enhance immunosuppression through a number of mechanisms involving the chronic inflammatory response and its mediators enhancing the effects of MDSCs and the polarization of other immune cells to a pro-tumorigenic state.^4^ To develop rational combination-treatment strategies, such as the addition of immunotherapy, we must first understand how the tumor–immune microenvironment in EC responds to chemotherapy and radiation. Hence, the purpose of this study was to define the changes within the tumor–immune microenvironment in patients with EC who have undergone neoadjuvant treatment and thus identify potential immune-related prognostic biomarkers.

## Methods

### Patient Cohorts

The patients in this study were identified through a retrospective database from two major metropolitan hospitals in Melbourne (Peter MacCallum Cancer Centre [PMCC] and St Vincent’s Hospital Melbourne [SVHM]) between 2010 and 2020. The discovery (PMCC) and validation (SVHM) cohorts included patients who were diagnosed with nonmetastatic (cT1-4, N0+), resectable EC who underwent neoadjuvant treatment (including chemoradiation or chemotherapy alone). Then, esophagectomy and clinicopathological characteristics were collected (Table 1). All of the patients in the cohort had preoperative staging with gastroscopy, CT, and PET-CT (with the exception of one patient whose data were not available). Ethnicity data were not routinely recorded in this retrospective database. Relevant ethics approval was granted by the PMCC and SVHM Human Research Ethics Committees (HREC numbers 10/108 and 18/211).

**Table 1:** Comparison of the clinicopthological charcteristics of the discovery and validation cohorts

Parameter	PMCC (n = 55)	SVHM (n = 59)	*p*-value
Age (median[range])	66 (46–79)	65 (36–78)	0.72
Histology			0.2
Adenocarcinoma	38 (69%)	47 (79.7%)	
Squamous cell carcinoma	17 (31%)	12 (20.3%)	
Grade			0.54
Well differentiated	1 (1.85°%)	1 (1.68%)	
Moderately differentiated	23 (41.8%)	27 (45.8%)	
Poorly differentiated	30 (54.5%)	27 (45.8%)	
Not available	1 (1.85%)	4 (6.72%)	
Baseline stage on PET-CT			0.07
Node negative	35 (63.6%)	27 (45.8%)	
Node positive	20 (36.4%)	31 (52.5%)	
Not available		1 (1.7%)	
Treatment modality			0.09
CROSS / Carboplatin/Cisplatin and 5FU + 50.4Gy	50 (90.9%)	47 (79.7%)	
Other (ECF / FLOT)	5 (9.1%)	12 (20.3%)	
Re-staging PET response			N/A
Complete metabolic response	19 (34.5%)	8 (13.5%)	
Partial metabolic response	34 (61.8%)	6 (10.2%)	
Not performed	2 (3.7%)	45 (76.3%)	
Tumour Regression Grade			0.79
TRG 1	15 (27.3%)	18 (30.5%)	
TRG 2–3	24 (43.6%)	22 (37.3%)	
TRG 4 -5	16 (29.1%)	19 (32.3%)	
Pathological nodal status			0.7
Node positive	26 (47.3%)	30 (50.8%)	
Node negative	29 (52.7%)	29 (49.2%)	

N/A, Not performed as a majority of patients in the validation cohort were not restaged using PET

### Evaluation of Transcriptomic Landscape with NanoString^TM^

The number of tumor sections available for review for each patient varied. All tumor sections were reviewed by a single pathologist in each cohort, blinded to survival data. The tumor section that was considered the most representative of the patient’s response to neoadjuvant treatment (i.e., the section with the most tumor by surface area) was used for RNA extraction and immunohistochemical analysis. RNA from formalin-fixed, paraffin-embedded (FFPE) pretreatment biopsies and posttreatment surgical resection specimens in esophageal adenocarcinoma (EAC) patients (n = 23) with a noncomplete pathological response (TRG 2-5) were hybridized with the NanoString PanCancer Immune Profiling Panel (NanoString Technologies Inc., Seattle, WA), consisting of 770 fluorescently tagged barcoded gene probes (Table S1) following recommended protocol (*Supplementary Methods*). Post-hybridisation, samples were transferred into the NCounter FLEX system and analysed with NCounter Digital Analyzer by setting the Fields of View count to 555. One common sample was included in every run as a control to account for batch effects.

### Immunohistochemistry and Cell Quantification

Tumor tissue was cut into 4µm sections and incubated with primary antibody (1:50 Dako monoclonal mouse anti-human CD15, M3631, Agilent Technologies; 1:80 polyclonal goat anti-human CXCL5, AF254, R&D Systems) for 60 min at room temperature (CD15) or overnight at 4 °C (CXCL5). Following primary antibody staining, sections were washed with phosphate-buffered saline (PBS) then incubated with secondary antibody (Dako anti-mouse HRP K4001 or horse anti-goat IgG ImmPRESS MP-7405-NB) for 10 min followed by DAB chromogen (Dako Liquid DAB+ Substrate Chromogen System, K3468) and counterstaining with hemotoxylin. Stained slides were digitally scanned using the Olympus VS120 Virtual Slide Microscope system and uploaded onto HALO Image Analysis Platform (v2.3, Indica Labs, Albuquerque, NM). Using a haemtoxylin and eosin (H&E) section regions of interest were marked, by the study pathologists (OP and TL), for cell quantification. Measurement parameters were optimised to ensure correct detection of nuclei and positive or negative DAB chromogen staining and each image was checked post-analysis to ensure accurate reproducibility across all samples.

In a separate analysis, tumor-infiltrating lymphocytes (TIL) and neutrophils were quantified in tumor specimens and were expressed as the total percentage area (tumor/scar and stroma) of infiltration according to an established method.^5^

### Tumor Inflammation Signature Score

The tumor inflammation signature (TIS) score was calculated based on the set of genes as derived by Ayers et al.^6^ The 18-gene, T-cell–inflamed gene expression profile was calculated by averaging the normalised linear counts of the constituent genes in the panel, with the exception of the genes *HLA-DRB1* and *NKG7* as they are not present in the PanCancer Immune Profile Panel.

### Neutrophil to Lymphocyte Ratio

Where available (n = 82), full blood examination results were obtained from the discovery and validation cohort of patients to calculate the neutrophil-to-lymphocyte ratio (NLR) post-neoadjuvant treatment. The NLR was calculated by dividing the absolute neutrophil count (measured in number of cells per liter of blood) by the absolute total lymphocyte count. Change in NLR (n = 44) was calculated from the paired baseline NLR counts obtained prior to the commencement of neoadjuvant treatment and post-neoadjuvant treatment NLR counts, which was measured at least 2 weeks after the cessation of neoadjuvant treatment.

### Statistical Analysis

Differences between clinicopathological characteristics in the discovery and validation cohorts were determined by using Fischer’s exact tests for categorical variables. Analysis of cell counts with nodal status, disease recurrence, and tumor inflammation signature (TIS) scores were performed by using Mann-Whitney *U* tests. For survival analyses, PFS was defined as the time from the start of treatment to clinical or radiological detection of recurrent disease, and used as the primary outcome. Patients were censored due to perioperative mortality or death where there was no documented recurrence of disease. Statistical analyses were performed using a combination of GraphPad Prism 9 (v9.1.2) and R statistical software (version 1.3.1056, Vienna, Austria).^7^ A two-tailed *p*-value < 0.05 was considered a statistically significant difference for all analyses.

### Creation and Validation of Nomogram

A Cox proportional hazards model using a combination of the variables (histological grade, TRG, and natural log transformed CD15^+^ cell count) was performed using the PMCC discovery cohort. Nodal status was included as a covariate in an earlier model; however, this variable resulted in an overfitted model (using model shrinkage) and did not significantly contribute toward the predictive power of the model. Thus, we excluded this covariate from the final model. Furthermore, due to the high proportion of missing neutrophil to lymphocyte ratio (NLR) values (32% missing), it was not included in the model. Proportionality assumption was checked in all of the survival analyses models using the Grambsch-Therneau test.^8^ No violations were found. A nomogram was constructed using the aforementioned covariates, and predictive accuracy and discrimination performance were assessed by using two measures of the model’s accuracy to determine PFS: the concordance probability estimate (CPE) and concordance index (C-index) where a score of 0.5 indicates inferior predictive capacity and 1 indicates a perfect model.^9^ To validate the model, the coefficients derived from the Cox model in the discovery cohort were used in the external validation cohort, and the CIs of C-index and CPE were estimated by bootstrapping with 1,000 resamples. Additional methods are provided in the supplementary material.

## Results

### Changes in the Immune-based Transcriptomic Profile with Neoadjuvant Chemoradiation

**Fig. 1:**
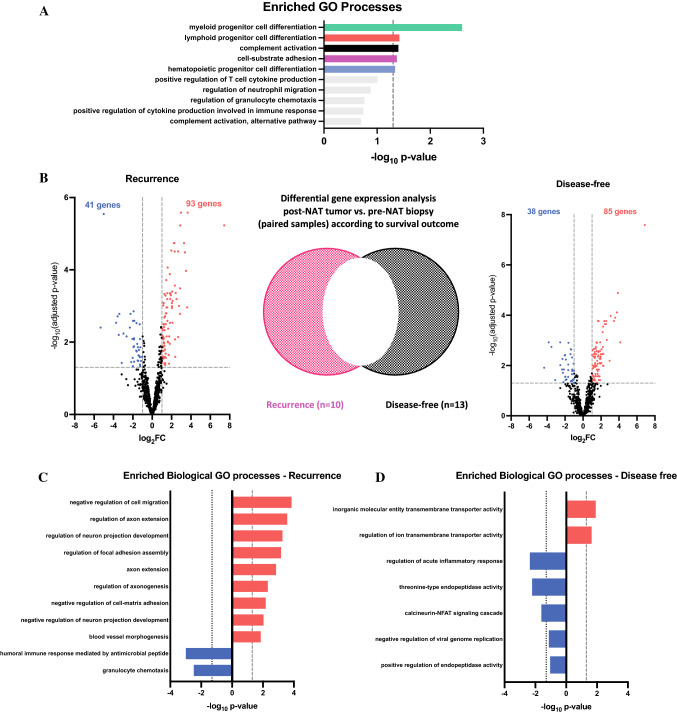
Changes in the transcriptomic profile of EC tumors with neoadjuvant treatment. (**A**) Gene enrichment analysis for immune-based and biological gene ontology (GO) processes for upregulated genes in all patients. Colored bars highlight significantly enriched processes and the broken vertical line denotes term adjusted *p*-value < 0.05. (**B**) Differential gene expression analysis of paired pre-treatment biopsies and posttreatment tumor surgical resection specimens between disease-free and recurrent disease patients at 2 years. (**C**) Gene enrichment analysis for significantly upregulated (red) and downregulated (blue) immune-based and biological GO processes post neoadjuvant treatment in patients with recurrent disease at 2 years and (**D**) in patients who are disease-free at 2 years

We used ClueGO^10^ to decipher the biological processes associated with significantly upregulated or downregulated genes (log_2_fold change $\ge$ ± 1 and adjusted *p*-value < 0.05) in patients with EAC. In pretreatment tissue, genes associated with T-cell chemotaxis and the humoral immune response were more highly expressed in patients who were disease-free at 2 years versus those who recurred (Fig. S2; Table S2). We then assessed the changes in immune-based transcriptomic profiles in paired pre- and posttreatment EAC tissues from 23 patients (TRG 2-5) to identify chemoradiation-induced changes in immune-related genes (Tables S3 and S4). Significantly upregulated, immune-related processes post-neoadjuvant treatment consisted of myeloid progenitor cell differentiation, lymphoid progenitor cell differentiation, complement activation, cell-substrate adhesion and hematopoietic progenitor cell differentiation (Fig. 1A; Table S5). Conversely, genes involved in T-cell proliferation and type 1 interferon-mediated signalling pathways were downregulated (Table S6).

Next, we compared the effect of neoadjuvant treatment on gene expression in patients who have recurrent disease within 2 years with those who are disease-free (Fig. 1B). The genes induced by neoadjuvant chemoradiation in patients that recur reveal processes that contribute to a pro-tumorigenic environment conducive for metastatic disease, such as negative regulation of cell-matrix adhesion and angiogenesis (Fig. 1C; Table S7). Importantly, genes belonging to the antimicrobial humoral immune response and granulocyte chemotaxis were downregulated following neoadjuvant therapy in patients with recurrent disease (Fig. 1C; Table S8). In patients who remain disease-free, genes related to membrane transporter activity were upregulated following NAT, whilst there were no significantly upregulated immune-related pathways (Fig. 1D; Table S9). In disease-free patients, genes related to the regulation of the acute inflammatory response, calcium signaling, and endopeptidase activity were downregulated (Fig. 1D; Table S10).

**Fig. 2:**
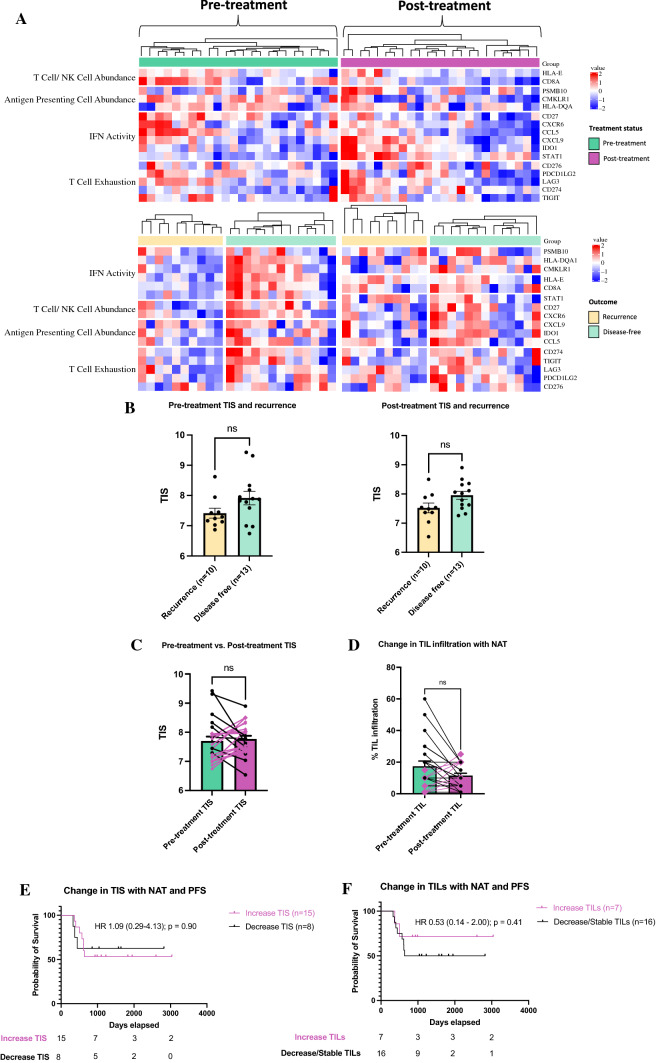
Effect of neoadjuvant treatment on adaptive immunity. (**A**) Expression profile of tumor inflammation signature (TIS)-based genes before and after treatment in disease-free and recurrent patients at 2 years. (**B**) Mean TIS scores in pre- and posttreatment tumor resection specimens (error bars represent standard error of the mean; ns: not significant). Paired pre- and posttreatment TIS scores (**C**) and TIL infiltration (**D**) (pink lines and symbols indicate patients whose scores increased). (**E+F**) Kaplan Meier survival curves (log rank test) of progression free survival (PFS) in patients with an increase TIS/TILs versus those with a decrease TIS/TILs

We then used the tumor inflammation signature score (TIS) to determine the effects of neoadjuvant treatment on adaptive immunity (Fig. 2A).^6^ Pretreatment and posttreatment TIS scores were not associated with disease-free survival at 2 years (Fig. 2B). In paired pre- and posttreatment samples, there was no difference in mean TIS score or mean TIL infiltration with neoadjuvant treatment (Fig. 2C, D). Furthermore, there was no survival advantage in patients who had an increase in TIS or TIL infiltration following neoadjuvant treatment (Fig. 2E, F).

Next, to decipher the type of myeloid cell that is affected by neoadjuvant treatment, we looked at the differentially expressed genes in posttreatment tumor resection specimens between patients with recurrence and those who are disease-free at 2 years (Fig. S3; Table S11). Expression of genes related to neutrophil function (*LCN2*^11^*, CD24*^12^*, F2RL1*^13^) were upregulated in posttreatment tumour specimens from patients who subsequently recurred. In contrast, expression of genes related to adaptive immunity were increased in tumours of patients who remained disease-free. However, there were no significant differences in relative immune cell proportions between recurrent and disease-free patients using the CIBERSORT algorithm (Fig. S4).^14^

### CD15^+^ Cell Infiltration in Partial Responders is Prognostic for Progression-free Survival

**Fig. 3:**
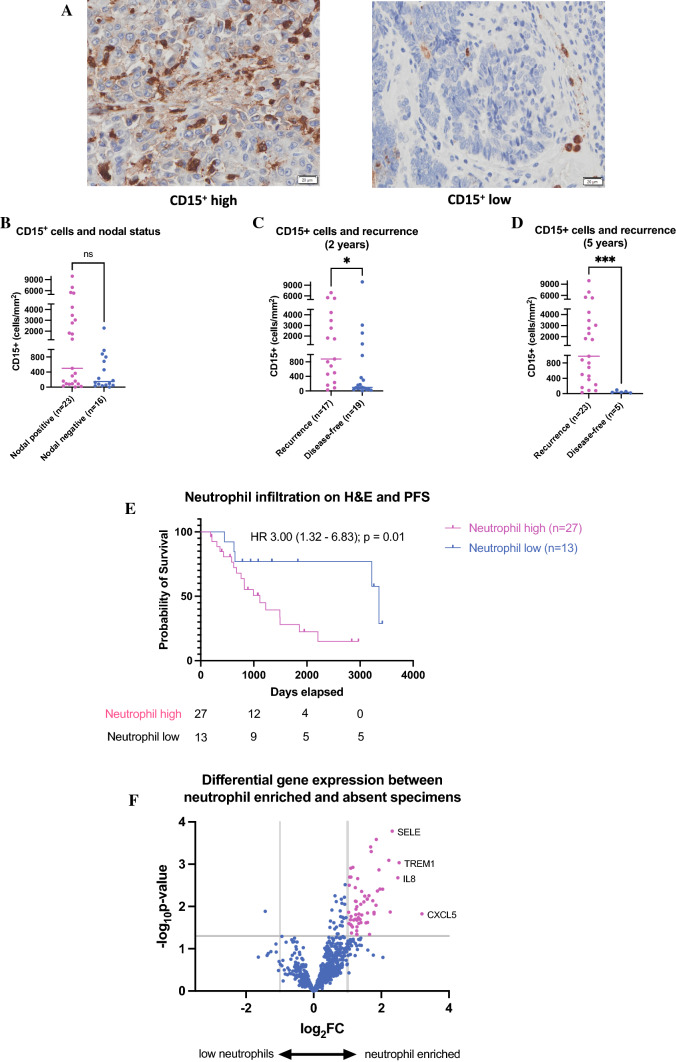
CD15^+^ cell infiltration post-neoadjuvant treatment is associated with inferior prognosis. (**A**) Representative tumor sections with high and low CD15^+^ cell infiltration. CD15^+^ cell counts of tumor regions were quantified using HALO imaging software and correlated to (**B**) pathological nodal status and (**C**) recurrence of disease at 2 years and at (**D**) 5 years posttreatment. (Note: one tumor section unable to be assessed due to tissue degradation) (*p < 0.05; ***p < 0.001.) (**E**) Kaplan-Meier survival curve (log-rank test) of progression-free survival in high ($\ge$ median) and low (< median) neutrophil infiltration groups. (**F**) Volcano plot of genes upregulated in patients with neutrophil-enriched tumor specimens vs. low-neutrophil infiltrated tumors post neoadjuvant treatment (vertical grey lines delineate log_2_ fold change of ±$1$ and horizontal grey line represents threshold of significance at *p*-value < 0.05)

We next sought to confirm the findings of neutrophil related genes in patients post-neoadjuvant therapy. Using the discovery cohort of patients with residual tumor following neoadjuvant treatment (TRG 2-5, n = 39; 1 patient sample unable to be assessed), we stained for CD15^+^ cells as a surrogate marker for tumor-infiltrating neutrophils (Fig. 3A).^15^ Although high CD15^+^ cell infiltration was not associated with nodal status at the time of resection (Fig. 3B), we did observe a significant association of higher CD15^+^ cell infiltration in the resected specimen from patients with recurrent disease at 2-year follow-up (Fig. 3C). This was even more pronounced at 5-year follow-up, where only those patients with the lowest CD15^+^ cell counts at resection were still disease-free (Fig. 3D). Furthermore, we observed a similar association of higher CD15+ cell infiltration with recurrent disease using only EAC samples (Fig. S6). To confirm that tumor-infiltrating neutrophils were responsible for this prognostic effect, tumor-infiltrating neutrophils were semiquantitatively scored on H&E sections and compared with their corresponding CD15^+^ section (Fig. S5). When the cohort was divided into high and low groups according to the median score for neutrophil infiltration as assessed by H&E, patients with neutrophil enriched tumors had inferior PFS (hazard ratio [HR] 3.00; 95% confidence interval [CI] 1.32–6.83]; *p* = 0.01) (Fig. 3E).

To investigate the potential mechanism(s) underlying the infiltration of neutrophils into the tumor microenvironment, we analyzed the differential expression of genes between posttreatment tumor specimens from neutrophil enriched and neutrophil low tumors (Figs 3F; Table S12). Genes involved in neutrophil trafficking (*IL8, CXCL5, SELE, TREM1*)^16,17^ were upregulated by greater than fourfold in neutrophil-enriched tumors. For the purposes of validation, we selected *CXCL5* due to its highest-fold change and performed IHC in posttreatment tumor specimens with residual pathological disease. CXCL5^+^ cell density correlated with CD15^+^ cell infiltration (Figs. S7A-B). High CXCL5^+^ cell counts also were significantly associated with pathological nodal disease (Fig. S7C).

**Fig. 4:**
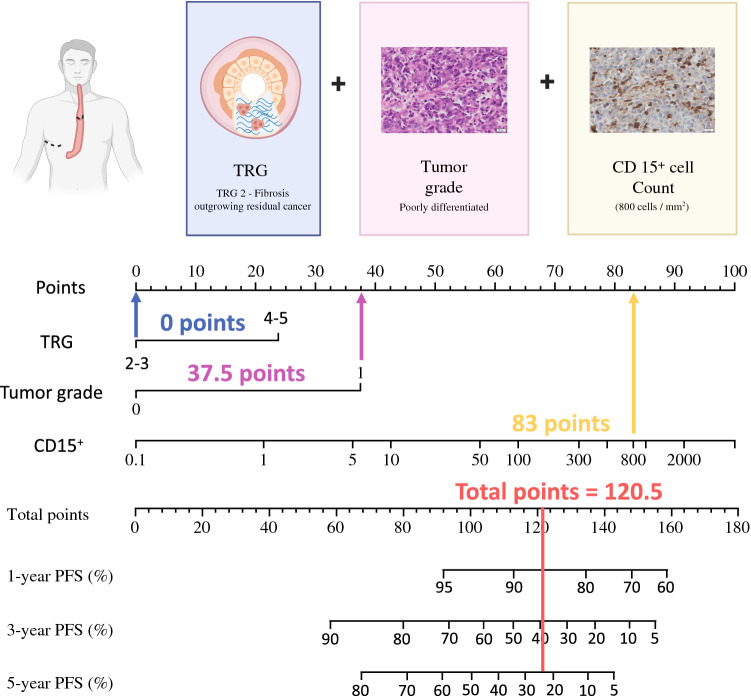
Nomogram for predicting progression free survival in esophageal cancer post-neoadjuvant treatment. The nomogram was developed using the PMCC training cohort with the covariates tumor regression grade (TRG 2-3; TRG 4-5), tumor histological grade (0: well to moderately differentiated; 1: poorly differentiated), CD15 count (cells/mm^2^). To use the nomogram, determine the sum of the scores for each variable. Find this number on the line for “Total points” and the vertical intersect with the lines for progression-free survival (PFS) represents the percentage chance of PFS. In the example provided in the top panel, the patient had a TRG of 2 (0 points), poorly differentiated tumor (37.5 points), and CD15+ count of 800 (83 points). Thus, this patient’s chances of being cancer free at 1-, 3-, and 5 years post treatment would be approximately 86%, 39% and 24% respectively

To assess the utility of CD15^+^ cell density as a potential biomarker, we compared its prognostic ability against other patient clinicopathological factors, such as tumor grade and tumor regression grade. Due to the limitations in the discovery cohort sample size, we were unable to incorporate additional known prognostic variables in our model. In a multivariable Cox proportional hazards model using these aforementioned variables, CD15^+^ cell infiltration was the most significant independent predictor for PFS (Table 2). We used these covariates to develop a nomogram (Fig. 4) with internal validation using the PMCC discovery cohort, resulting in a C-index of 0.80 (95% CI 0.68–0.9) and a CPE of 0.77 (95% CI 0.69–0.86), which indicates that our model has high discriminatory ability. Using a calibration curve, the performance curve of our prognostic model using CD15^+^ count was more accurate than the same model incorporating nodal status as a variable instead of CD15^+^ (Fig. S8). Applying the nomogram to the independent validation cohort (SVHM), the predictive performance of the model resulted in a C-index of 0.81 (95% CI 0.69–0.91) and a CPE of 0.78 (95% CI 0.7–0.86).

**Table 2:** Univariate and mulitvariate Cox proportional hazards model of clinicopathological predictors for progression

		Univariate analysis		Multivariate analysis	
Parameter		HR (95% CI)	*p*-value	HR (95% CI)	*p*-value
Age	≥ 66 versus <66 (median)	1.20 (0.53–2.74)	0.66		
Histological grade	Poorly differentiated versus well to moderately differentiated	4.28 (1.33–12.68)	**0.01**	3.72 (1.23–11.2)	**0.02**
Pathologic Nodal Status	Nodal positive versus negative	2.45 (0.99–6.07)	0.05		
TRG	4–5 versus 2–3	2.27 (0.95–45.41)	0.06	2.29 (0.93–5.6)	0.07
CD15+ cells	log (CD15)*	5.76 (2.03–16.3)	**< 0.01**	5.55 (1.94–15.8)	**< 0.01**

*p*-values < 0.05 are given in bold

TRG, Tumour regression grade

*Continuous variable

### High Neutrophil-to-Lymphocyte Ratio Post-neoadjuvant Treatment is Associated with Disease Recurrence

**Fig. 5:**
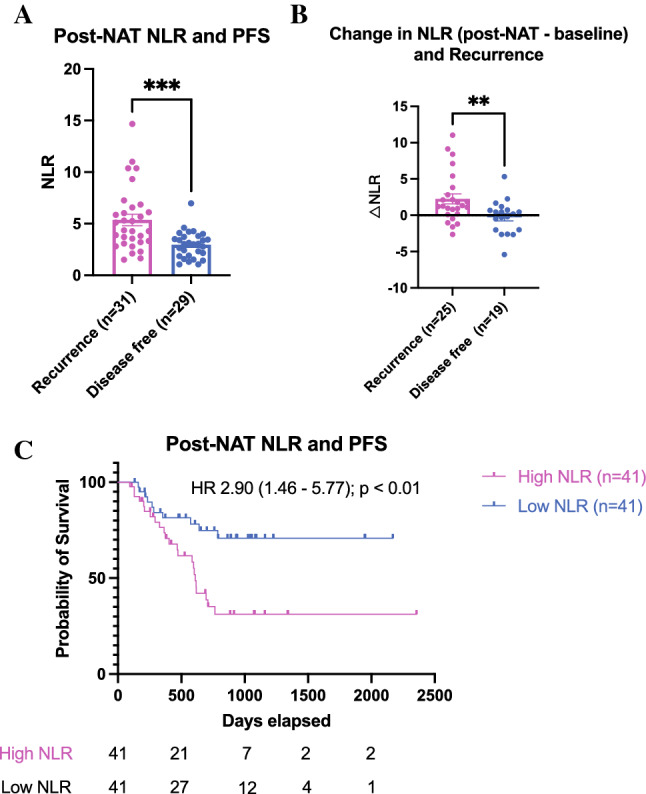
Post-neoadjuvant treatment (NAT) neutrophil to lymphocyte ratio (NLR) is associated with CD15^+^ infiltration and prognosis. (**A**) Posttreatment NLR and status of disease recurrence at 2 years. (**B**) Change in NLR with neoadjuvant treatment (posttreatment NLR – baseline NLR) and status of disease recurrence at 2 years. (**C**) Kaplan-Meier curve post-neoadjuvant treatment NLR and progression free survival in patients with low and high NLR (according to median) post-neoadjuvant treatment

Collectively, the data presented above implicate inflammation in the resected tumor as an indicator of future disease recurrence. The neutrophil-to-lymphocyte ratio (NLR) in patients’ blood samples is a surrogate marker of systemic inflammation and the balance between chronic inflammation and adaptive immunity.^18^ We therefore investigated the association between posttreatment NLR and disease recurrence (for this analysis we combined the discovery and validation cohorts due to a substantial proportion of missing NLR values; see Supplementary Table S13). Patients with recurrent disease at 2 years posttreatment had higher mean posttreatment NLR value compared with patients who were disease-free (5.37 vs. 2.96) (Fig. 5A). When we calculated the change in NLR between pre- and posttreatment, patients who recurred within 2 years after treatment had a mean NLR increase of 2.26 compared with minimal change in those that remained disease-free at 2 years (Fig. 5B). Using the median NLR value to dichotomize the entire cohort into “NLR high” (>3.46) and “NRL low” (<3.46) groups, patients with high NLR posttreatment are at high risk of relapse within 2 years with a median PFS of 611 days compared with more than half of “NLR low” patients still disease-free at this point (HR 2.90; 95% CI 1.46–5.77; *p* < 0.01; Fig. 5C). In a subgroup analysis, patients with both high CD15^+^ cell count and high NLR are 11 times more likely to have early disease recurrence (Fig. S9). These results point toward the possibility of using NLR as an accessible biomarker for predicting early disease recurrence post-neoadjuvant treatment.

## Discussion

Our study demonstrated that neoadjuvant treatment of EC results in an increase in expression of genes related to myeloid progenitor cell differentiation and showed that an increase in neutrophil infiltration is associated with recurrent disease. Neutrophils and MDSCs are implicated in the tumorigenesis and inflammatory microenvironment of EAC.^19^ Our findings suggest that, in EC, dynamic changes in the tumor microenvironment induced by neoadjuvant chemoradiation are complex and may be contributing to the progression toward metastatic disease.

In tumors with an immune microenvironment that is infiltrated by MDSCs, chemotherapy and radiation may establish a chronic inflammatory response and increase immunosuppression.^4^ This is especially pertinent in the setting of gastroesophageal junctional cancers whereby repeated bile-acid reflux has been shown to induce a chronic inflammatory response mediated by prostaglandin E2, which has been established as a key signaling molecule in inflammation-induced immunosuppression.^20,21^ To our knowledge, our study is the first to establish a link between tumor-infiltrating neutrophils induced by neoadjuvant chemoradiation and its association with inferior prognosis.^16^ Further evidence that complements these findings is the observation that elevated posttreatment NLR, which is a marker of systemic inflammation, also is an indicator of poor prognosis. More importantly, it is possible that chemoradiation exacerbates this inflammatory response as evidenced by the increase in NLR posttreatment compared with pretreatment levels in patients with recurrent disease. Hence, these results raise the possibility of using NLR after completion of neoadjuvant treatment as a convenient and easily accessible biomarker to identify patients at high risk of recurrence *before* surgery. Patients who are at high risk of disease relapse may benefit from additional perioperative treatment or, in some cases, surgical resection may not be appropriate.

Our results are consistent with a large meta-analysis of gene expression signatures obtained from more than 18,000 pan-cancer solid organ biopsy samples, which demonstrated a predominance of neutrophil gene expression signatures that were associated with inferior prognosis.^22^ Critically, estimated neutrophil cell proportions using the CIBERSORT algorithm did not correlate with the content of necrotic tissue, thus suggesting that the presence of neutrophils is more than just the “bystander effect” secondary to inflammation. Interestingly, previous work by Park et al. also demonstrated that neoadjuvant chemoradiation in ESCC was associated with an increase in neutrophil gene signatures using the CIBERSORT algorithm.^23^ However, this finding was not validated by using immunohistochemistry and its association with treatment response or prognosis was unclear.

A limitation of our study, which also is a possible direction for future work, is the lack of spatial correlation with the transcriptomic data and the use of a single immunohistochemical marker to identify neutrophils. Histologically, neutrophils and polymorphonuclear MDSCs are indistinguishable.^24^ What may be more insightful is to determine the transcriptomic and proteomic profiles of different classes of neutrophils through the use of digital spatial profiling and single cell RNA sequencing.^25^ This will confirm whether these neutrophils are purely bystanders of the inflammatory response induced by neoadjuvant chemoradiation or truly exert immunosuppressive effects, whereby, consistent with the current literature it would be more appropriate to describe these cells as polymorphonuclear MDSCs.^26^ In addition, it may verify our observations of immune independent tumor-intrinsic processes, which are responsible for the formation of metastatic disease.

Further limitations are the small sample size of the discovery cohort, missing NLR data, and the heterogenous follow-up times. Furthermore, the inclusion of additional covariates within the model with a small sample size risks overfitting the model, which results in a model with suboptimal prognostic performance. These limitations precluded our ability to incorporate NLR and pathological nodal status into our prognostic model. Future work in a larger cohort of patients will be required to refine our model with the addition of these variables. Nevertheless, an advantage of our study is the key finding that tumor infiltrating neutrophils is the most significant predictive variable along with the validation of our model using an independent cohort.

Overall, our study raises the possibility that neutrophils maybe implicated in the promotion of metastatic disease within EC patients treated with neoadjuvant chemoradiation.

## Conclusions

Our study provides evidence that an increase in neutrophils following neoadjuvant chemoradiation is associated with poor outcome in EC and that systemic NLR is a potential biomarker predictive of future disease recurrence.

## Supplementary Information

Below is the link to the electronic supplementary material.

Supplementary file1 (DOCX 28 KB)

Supplementary file2 (PDF 2412 KB)

Supplementary file3 (XLSX 261 KB)

## Data Availability

Nanostring nCounter data consisting of raw and normalised linear counts will be available upon reasonable request. Please submit a request to the corresponding authors, Associate Professor Nicholas Clemons or Associate Professor Cuong Duong.
