# Supplementary material for: Tumor-Infiltrating Neutrophils after Neoadjuvant Therapy are Associated with Poor Prognosis in Esophageal Cancer
Source: Ann Surg Oncol. 2022 Oct 2;30(3):1614–25. doi: 10.1245/s10434-022-12562-5 (PMC9908700; doi:10.1245/s10434-022-12562-5)
Supplement: Supplementary file 1 — Supplementary file1 (DOCX 28 KB) [file 10434_2022_12562_MOESM1_ESM.docx]

**Supplementary methods**

*Macro-dissection and RNA extraction*

Formalin-fixed and paraffin embedded (FFPE) pre-treatment biopsy and post NAT surgical resection specimens were obtained from patients within the cohort. The sections used for RNA extraction were evaluated by a specialist gastro-intestinal pathologist (OP). Sections that had the highest content of tumor or ‘scar’ by total surface area of the entire section were selected for RNA extraction. If the percentage of tumor or ‘scar’ was less than 50 percent of the entire surface area, these areas were marked by the pathologist on H&E section and non-tumor areas on sequential unstained sections were macro-dissected. Otherwise, FFPE sections 10 microns in thickness were cut from sections that did not require macro-dissection.

Deparaffinisation was performed with 500 µl of heptane in a 1.5 ml microfuge tube. FFPE sections were immediately placed in 1.5 ml microfuge tubes and processed on the day of sectioning or within 24 hours whilst remaining in 4^o^C storage. For samples that required macro-dissection, 5-micron thick sections were cut onto slides. As per the Nanostring Best Practice Guide for FFPE samples (LBL-10504-01 Sept 2017), the recommended minimum tissue surface for 5-micron thickness slides is 48 mm^2^ for RNA input. Thus, for each sample needing to be macro-dissected, the minimum number of slides were cut according to the recommendations established by Nanostring^TM^ . For each macro-dissected section, the tip of a fresh scalpel blade was used to carefully scrape the area of interest into a microfuge tube filled with 500 µl of heptane.

RNA extraction was performed using the RNeasy FFPE kit (Qiagen, Germany) following the manufacturer’s protocol with the following key changes: 240 µl of Buffer PKD was added to the microfuge tube; 60 µl of Proteinase K was added to the pellet; incubation time in proteinase K for 6 hours at 56^o^C;

*Assessment of RNA quality with TapeStation Analysis software and NanoString sample preparation*

RNA extracted from FFPE samples was analysed for concentration and size of RNA fragments using a TapeStation (Agilent Technologies, Santa Clara, USA) and examined for quality and quantity using TapeStation Analysis Software A.02.01 (Agilent Technologies, Santa Clara, USA). We used the concentration of RNA fragments above 200 nucleotides (DV_200_) as the basis for calculating input for NanoString (Supplementary Figure S1) using the DV_200_ concentration of 22.1 ng/µl. Therefore, the appropriate input accounting to DV_200_ is estimated by the following equation: (100/percent of sample > 200 nt) x 100 ng.

After prior optimisation work, target RNA input (accounting for DV_200_) was set at **200 ng** with a total input volume of 5 µl. If samples were too dilute, they were concentrated to approximately half their volume using a SpeedVac Vacuum Concentrator (Thermo Fisher Scientific, Waltham Massachusetts, USA). Subsequent sample preparation workflow was in accordance to the standard NanoString three-day sample preparation workflow.

*Statistical analysis*

To assess the prognostic effect of neutrophil infiltration with H&E staining, the cohort was divided into two groups according to the median count. Kaplan-Meier survival curves (log-rank test), hazard ratios and the corresponding 95% confidence intervals (CI) were calculated for the relationship between the magnitude of neutrophil infiltration and NLR with PFS. Pearson correlation and linear regression were performed to assess the relationship between cell types. Using the PMCC cohort, a nomogram was created using a combination of the variables: histologic histological grade, TRG, and natural log transformed CD15^+^ cell count.

*Statistical analysis of gene expression data*

Gene expression data from NanoString was analyzed using the *limma* package^2^ with R-software. Raw count data was normalised against the geometric mean of a set of housekeeping genes in the PanCancer Immune Panel gene set (Table S1) and also using common samples across each Nanostring batch run to account for any batch effects. Tumor samples with gene probe counts lower than the median for house-keeping genes after normalisation were excluded from analysis. Gene expression heat maps were generated using the *limma* package and volcano plots using linear normalised counts with GraphPad Prism 9. Due to the small sample size and exploratory nature of this study, genes that were differentially expressed with a log_2_ fold change of ≥ 1 or $\leq$ -1 and a nominal p-value < 0.05 were determined to be biologically significant.

For gene enrichment analysis and the generation of annotated functional networks, we used the Cytoscape application (v3.8.0) with ClueGO^3^ (v2.5.8) plugin. For this analysis, we used differentially expressed genes from the following analysis groups: 1) pre-treatment versus post-treatment in disease-free patients and 2) pre-treatment versus post-treatment in patients with recurrent disease. Importantly, the list of genes from the Nanostring PanCancer Immune Profiling panel was used as the custom reference set. Gene Ontology (GO) pathways were considered significant (adjusted p < 0.05) after correcting for multiple comparisons using Bonferoni’s test.

**References:**

1. Ayers M, Lunceford J, Nebozhyn M, Murphy E, Loboda A, Kaufman DR, et al. IFN-γ–related mRNA profile predicts clinical response to PD-1 blockade. J Clin Invest. 2017;127(8):2930–40.

2. Ritchie ME, Phipson B, Wu D, Hu Y, Law CW, Shi W, et al. limma powers differential expression analyses for RNA-sequencing and microarray studies. Nucleic Acids Res. 2015;43(7):e47–e47.

3. Bindea G, Mlecnik B, Hackl H, Charoentong P, Tosolini M, Kirilovsky A, et al. ClueGO: a Cytoscape plug-in to decipher functionally grouped gene ontology and pathway annotation networks. Bioinformatics. 2009;25(8):1091–3.
