# Supplementary material for: Tumor-Infiltrating Neutrophils after Neoadjuvant Therapy are Associated with Poor Prognosis in Esophageal Cancer
Source: Ann Surg Oncol. 2022 Oct 2;30(3):1614–25. doi: 10.1245/s10434-022-12562-5 (PMC9908700; doi:10.1245/s10434-022-12562-5)
Supplement: Supplementary file 2 — Supplementary file2 (PDF 2412 KB) [file 10434_2022_12562_MOESM2_ESM.pdf]

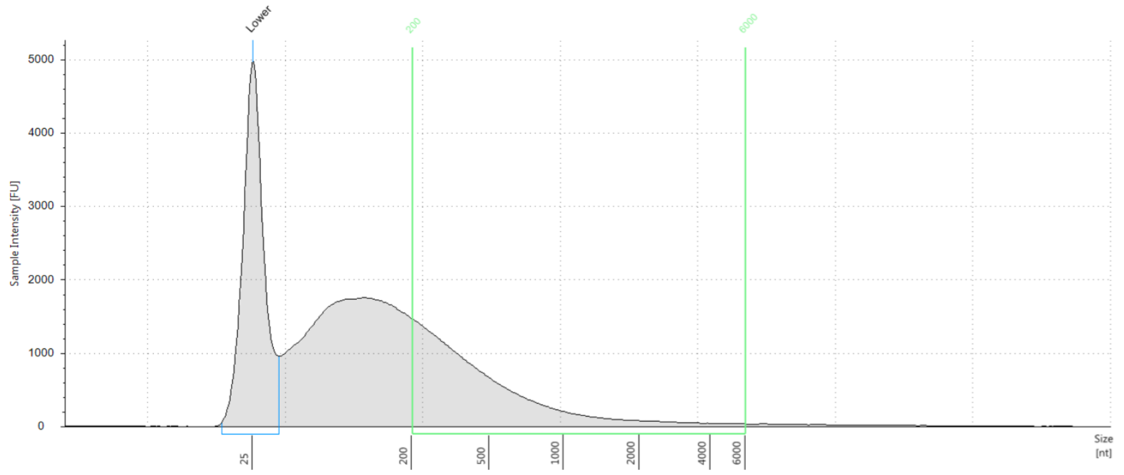


**Region Table**

| From [nt] | To [nt] | Average Size [nt] | Conc. [ng/µl] | Region Molarity [nmol/l] | % of Total | Region Comment | Color |
|---|---|---|---|---|---|---|---|
| 200 | 6000 | 1401 | 22.1 | 46.4 | 38.11 | | |

**Figure S1** – Tapestation analysis of input RNA sample for Nanostring workflow. The region gated by the green open rectangle demonstrates the proportion of sample containing RNA fragments greater than 200 nucleotides (nt) ($DV_{200}$).

A)

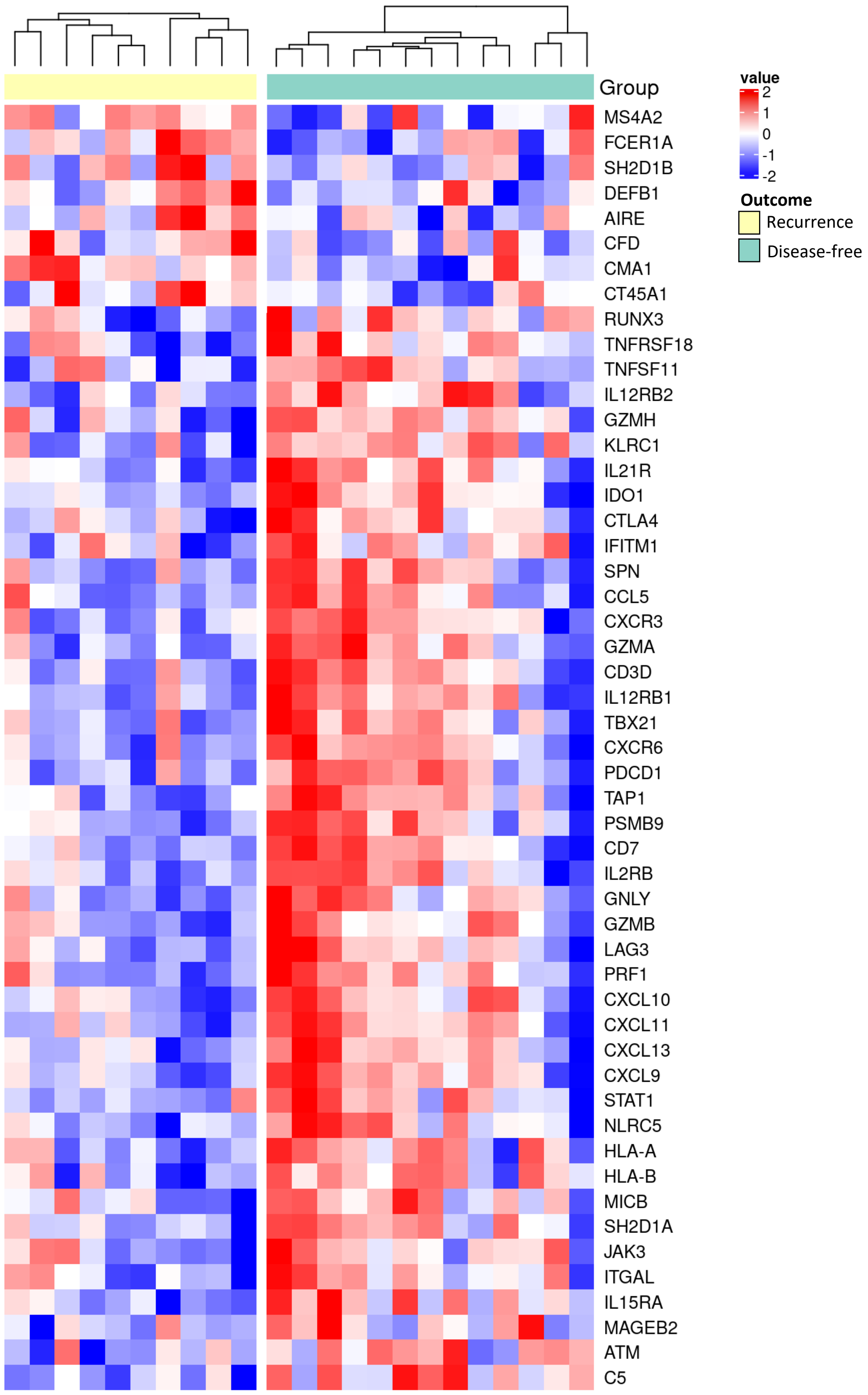


B)

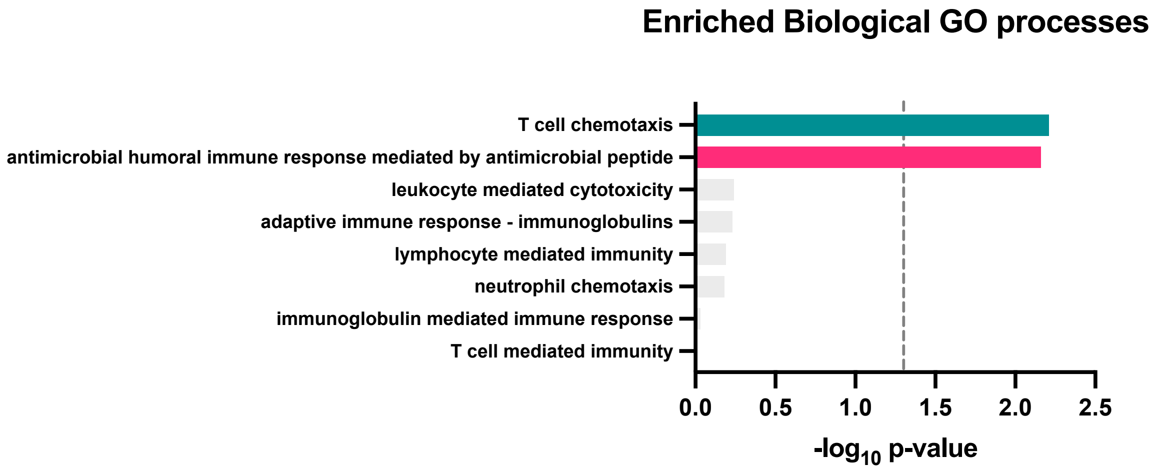


**Figure S2 –** Immune landscape of pre-treatment tumor specimens. (A) Differentially expressed genes (adj p-value < 0.05) in pre-treatment tumor samples between patients who are disease-free versus those with recurrent disease at 2 years. (B) Enrichment analysis for immune-based and biological gene ontology (GO) processes for upregulated genes in patients who are disease-free. Colored bars highlight significantly enriched processes and the broken vertical line denotes term adjusted p-value < 0.05.

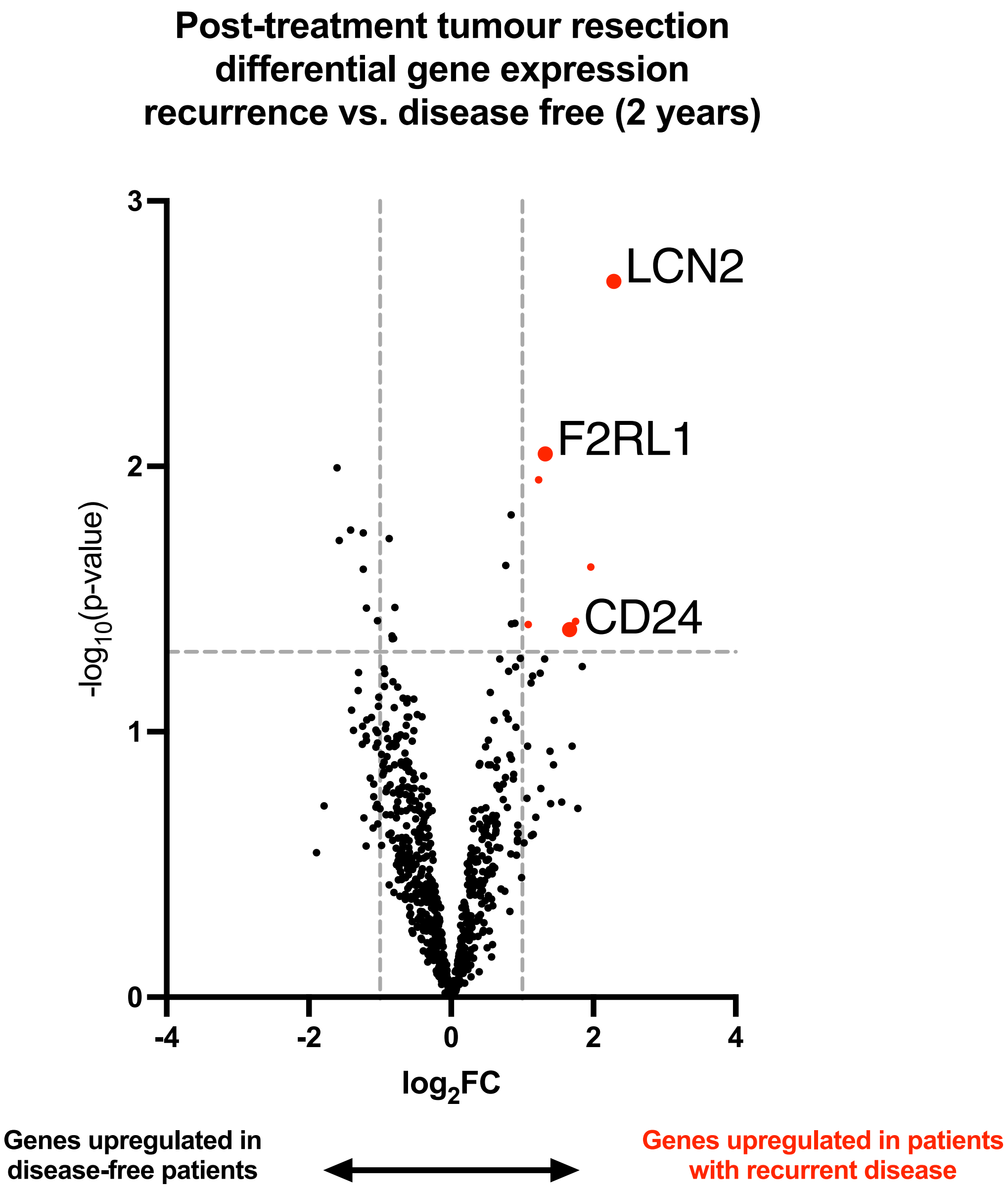


**Figure S3 -** Volcano plot showing differentially expressed genes in post-neoadjuvant tumor specimens in patients who went on to have recurrent disease compared to those who are disease free two years post-treatment. Genes highlighted in red are increased in patients with recurrent disease and are associated with neutrophil function. Horizontal grey line represents threshold of significance at $p$-value < 0.05. Vertical lines represent $\log_2$ fold change (FC) = $\pm 1$.

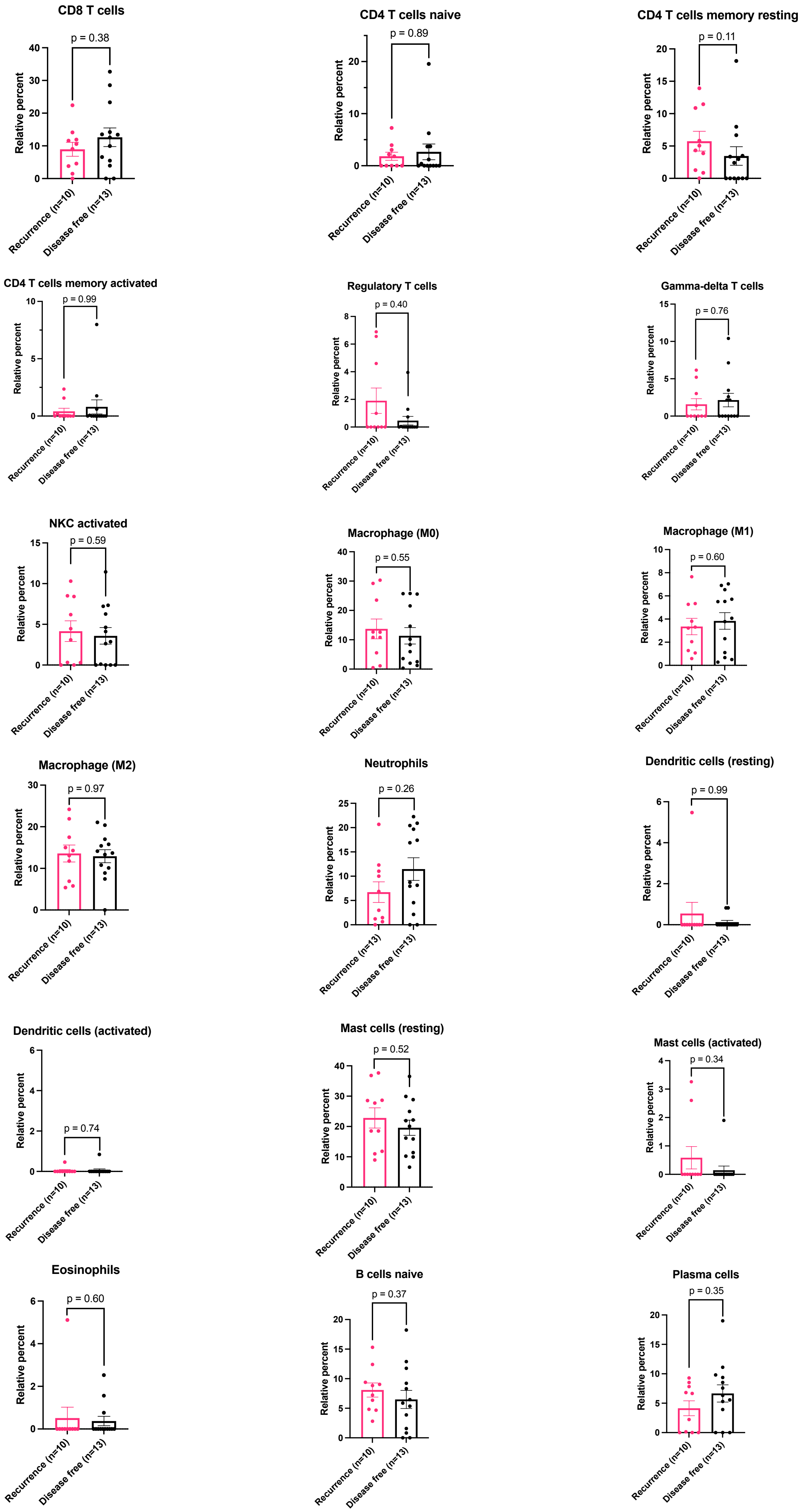


**Figure S4 –** Relative immune cell proportions in post-neoadjuvant treatment surgical resection specimens using the CIBERSORT algorithm. Differences in immune cell proportions were examined between patients with recurrent disease and those that remained disease free. Comparison of the two patient groups was performed using the Mann-Whitney U Test. (NS: not significant).

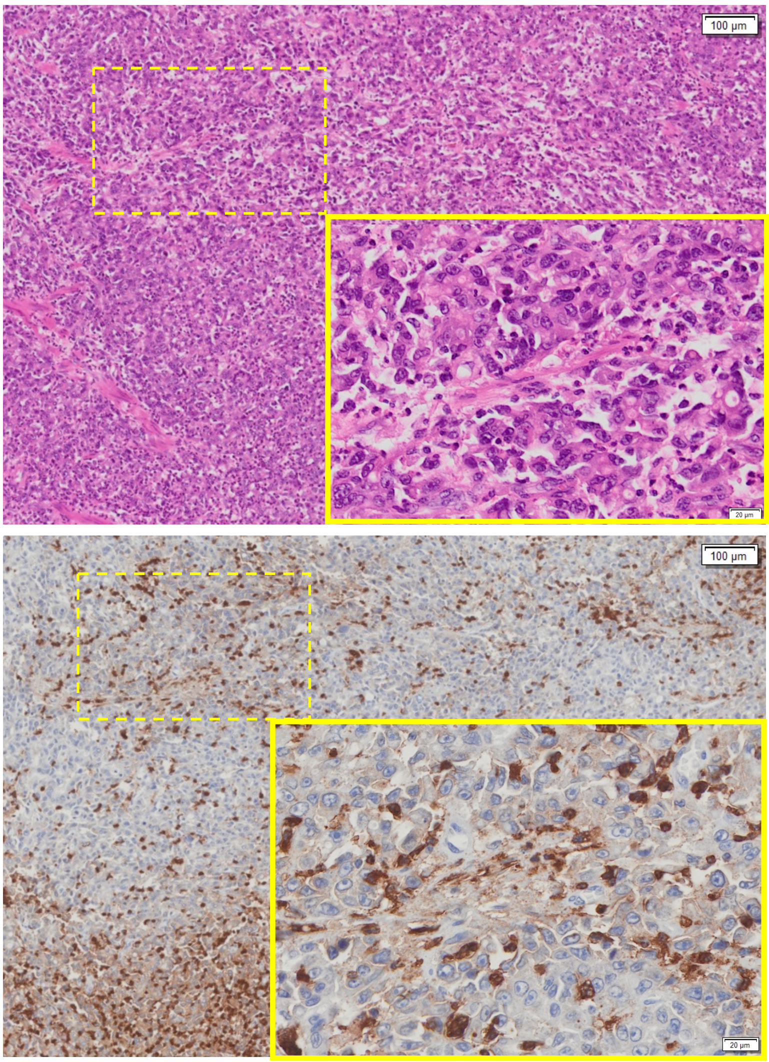


**Figure S5 –** Neutrophil enriched tumors post neoadjuvant treatment are associated with inferior prognosis. Tumor infiltrating neutrophils were identified and quantified using consecutive sections stained with H&E and anti-CD15 antibody. Shown is a representative example of a H&E and corresponding CD15 stain (insert is enlargement of boxed region). Scale bar = 100µm in low magnification image and 20µm in enlarged image.

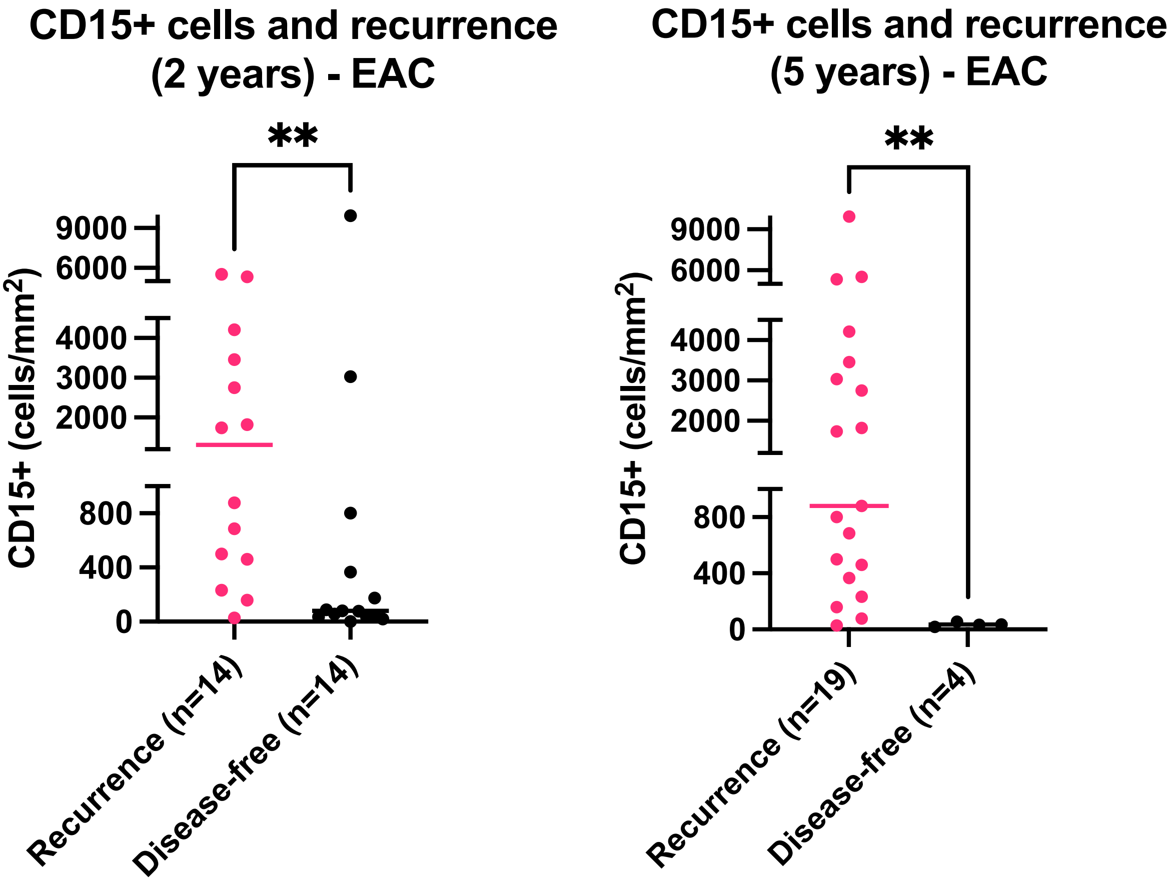


**Figure S6 –** CD15⁺ cell infiltration post neoadjuvant treatment is associated with inferior prognosis in patients with esophageal adenocarcinoma (EAC). CD15⁺ cell counts of tumor regions were quantified using HALO imaging software and correlated to recurrence of disease at (A) 2 years and at (B) 5 years post-treatment (Note: one tumor section unable to be assessed due to tissue degradation) (**p<0.01).

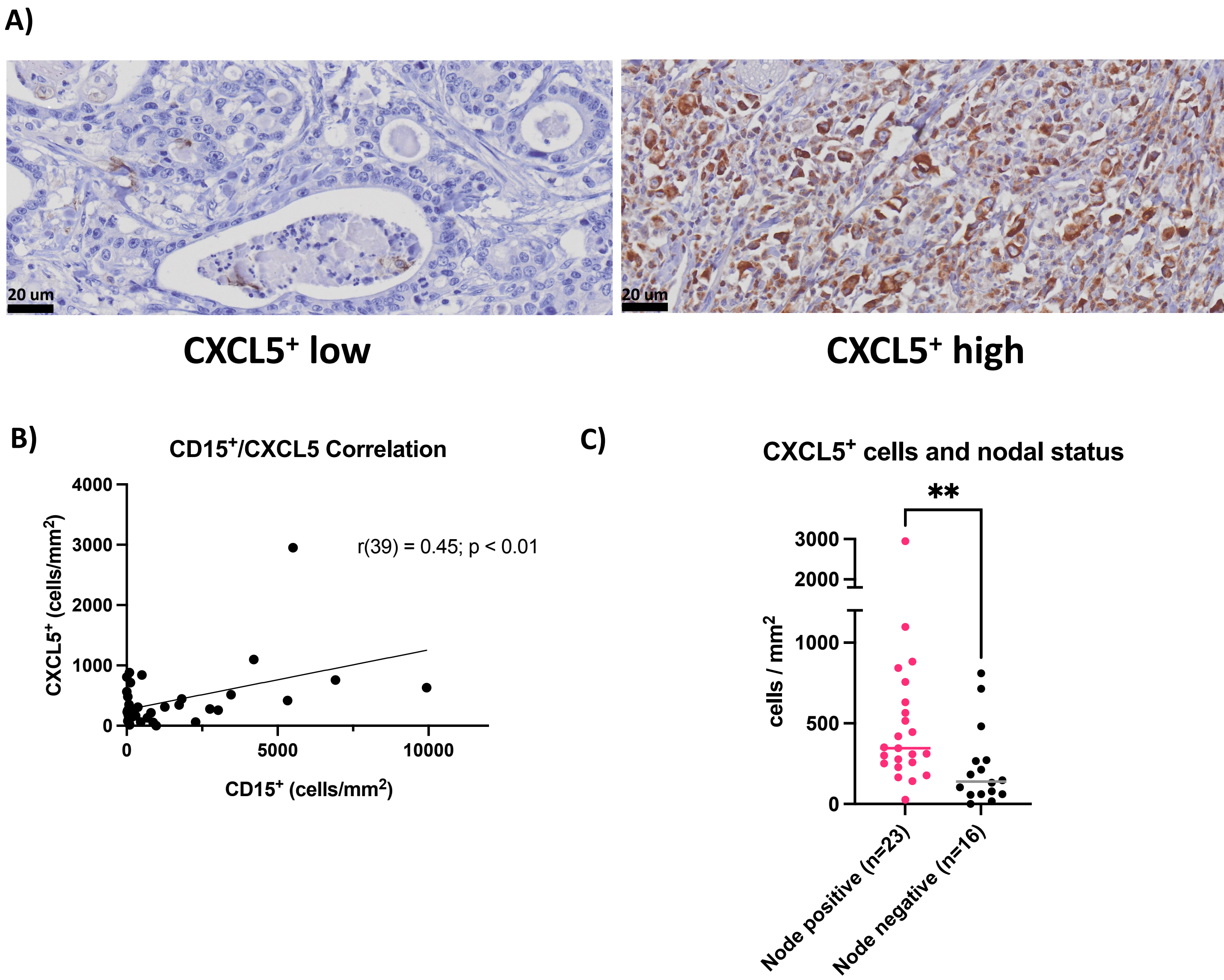


**Figure S7 –** The magnitude of tumor/stromal cell CXCL5$^+$ staining post neoadjuvant treatment is associated with nodal positivity and CD15$^+$ infiltration. (A) Post neoadjuvant treatment tumor sections of similar cellularity were stained with anti-CXCL5 antibody (brown) and counter stained with hematoxylin (blue). Shown are representative images of tumors with low and high CXCL5 staining. (B) The number of CXCL5$^+$ cells is associated with nodal positive disease. (C) The number of CXCL5$^+$ cells is significantly correlated with the number of CD15$^+$ cells.

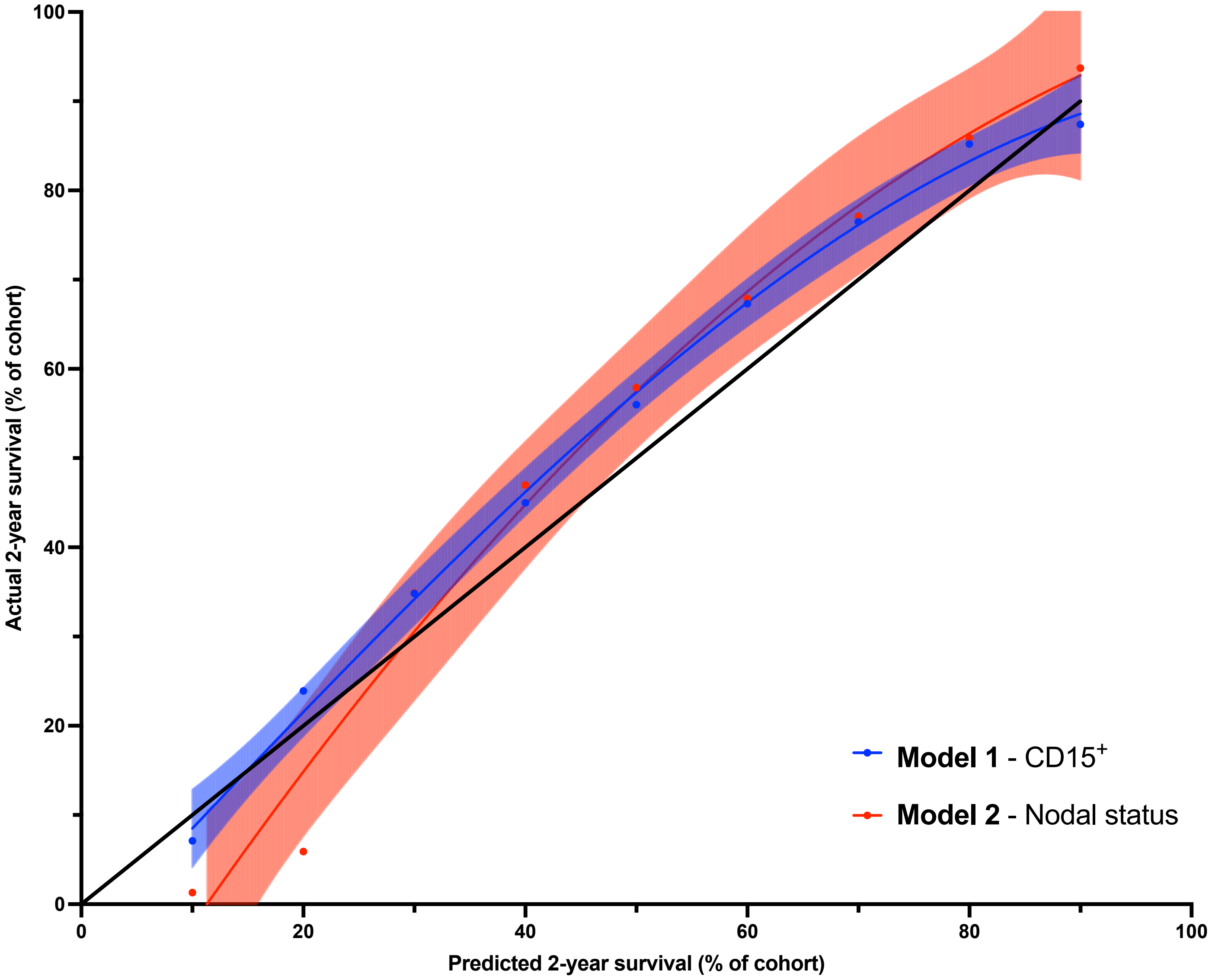


**Figure S8 –** Comparison of model accuracy using a calibration curve. The solid black line represents a perfect model (ie. C-index or concordance probability estimate [CPE] score of 1). The blue line represents the line of best-fit of a model using $CD15^+$ while the red line represents a model using pathological nodal status. The colored shaded regions represent the 95% CI of each model.

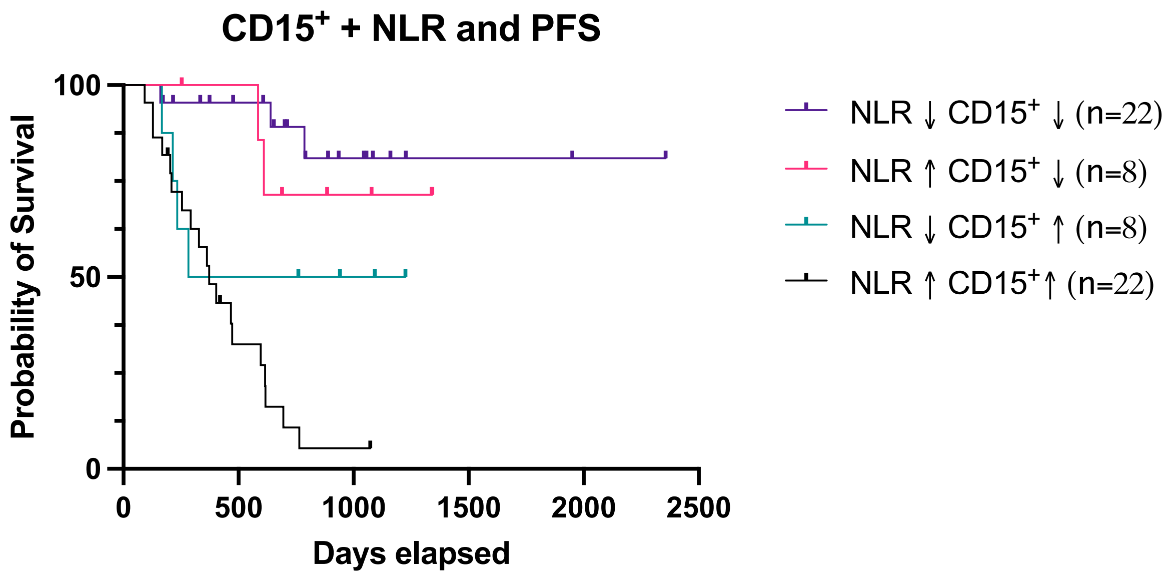


**NLR↑CD15$^+$↑** vs **NLR↓CD15$^+$↓** HR 11.85 (4.93 - 28.47) $p < 0.01$

**NLR↓CD15$^+$↑** vs **NLR↓CD15$^+$↓** HR 4.24 (0.75 - 24.00) $p = 0.02$

**NLR↑CD15$^+$↓** vs **NLR↓CD15$^+$↓** HR 1.76 (0.25 - 12.50) $p = 0.53$

**Figure S9** – Kaplan Meier survival curves using log-rank tests of progression free survival (PFS) at 2 years in patients with both neutrophil to lymphocyte ratios (NLR) and CD15$^+$ counts. Values in parentheses denotes the 95% CI.
